# Clinical Outcomes of COVID-19 Infection in Pregnant and Nonpregnant Women: Results from The Philippine CORONA Study

**DOI:** 10.3390/vaccines11020226

**Published:** 2023-01-19

**Authors:** Adrian I. Espiritu, Sybil Lizanne R. Bravo, Hannah Andrea A. Sombilla, Ourlad Alzeus G. Tantengco, Marie Charmaine C. Sy, Alvin Duke R. Sy, Veeda Michelle M. Anlacan, Roland Dominic G. Jamora

**Affiliations:** 1 Department of Clinical Epidemiology, College of Medicine, University of the Philippines Manila, Manila 1000, Philippines; 2 Department of Neurosciences, College of Medicine and Philippine General Hospital, University of the Philippines Manila, Manila 1000, Philippines; 3 Division of Neurology and Department of Psychiatry, University of Toronto, Toronto, ON M5S, Canada; 4 Department of Obstetrics and Gynecology, College of Medicine and Philippine General Hospital, University of the Philippines Manila, Manila 1000, Philippines; 5 Department of Obstetrics and Gynecology, Manila Doctors Hospital, Manila 1000, Philippines; 6 Department of Obstetrics and Gynecology, Medical Center Manila, Manila 1000, Philippines; 7 Department of Physiology, College of Medicine, University of the Philippines Manila, Manila 1000, Philippines; 8 Department of Biology, College of Science, De La Salle University, Manila 1004, Philippines; 9 Department of Epidemiology and Biostatistics, College of Public Health, University of the Philippines Manila, Manila 1000, Philippines; 10 Institute for Neurosciences, St. Luke’s Medical Center, Quezon City 1112, Philippines

**Keywords:** COVID-19, pregnancy, mortality, respiratory failure, ICU admission

## Abstract

Objective: Our study determined the association of pregnancy with various clinical outcomes among women with COVID-19 infection. Methods: We conducted a retrospective, cohort, subgroup analysis of the Philippine CORONA Study datasets comparing the clinical/neurological manifestations and outcomes of pregnant and nonpregnant women admitted in 37 Philippine hospitals for COVID-19 infection. Results: We included 2448 women in the analyses (322 pregnant and 2.126 nonpregnant). Logistic regression models showed that crude odds ratio (OR) for mortality (OR 0.26 [95% CI 0.11, 0.66]), respiratory failure [OR 0.37 [95% CI 0.17, 0.80]), need for intensive care (OR 0.39 [95% CI 0.19, 0.80]), and prolonged length of hospital stay (OR 1.73 [95% CI 1.36, 2.19]) among pregnant women were significant. After adjusting for age, disease severity, and new-onset neurological symptoms, only the length of hospital stay remained significant (adjusted OR 1.99 [95% CI 1.56,2.54]). Cox regression models revealed that the unadjusted hazard ratio (HR) for mortality (HR 0.22 [95% CI 0.09, 0.55]) among pregnant women was statistically significant; however, after adjustment, the HR for mortality became nonsignificant. Conclusion: We did not find a significantly increased risk of mortality, respiratory failure, and need for ICU admission in pregnant women compared with nonpregnant women with COVID-19. However, the likelihood of hospital confinement beyond 14 days was twice more likely among pregnant women than nonpregnant women with COVID-19.

## 1. Introduction

The coronavirus disease 2019 (COVID-19) has affected millions of people worldwide. Although a large amount of information on its risk factors, mode of transmission, clinical course, and treatment have been published, aspects of its disease course need investigation, particularly in the pregnant population.

A previous study noted that the most frequent symptoms in pregnant women with COVID-19 were cough (65%), fever (57%), shortness of breath (47%), sore throat (22%), anosmia (16%), and headache (15%) [1]. Since typical pregnancy symptoms may overlap with COVID-19 manifestations (e.g., shortness of breath, fatigue, nasal congestion, or nausea and vomiting), the clinician should be more discerning and thorough in evaluating the gravida during a pandemic.

Earlier reports in the Philippines indicated that several pregnant COVID-19 patients were asymptomatic, ranging from 22 to 42% of individuals [2,3,4]. Previous studies have also suggested that pregnant women with COVID-19 have a favorable clinical course compared with the nonpregnant population [2,5,6,7,8,9,10]; however, pre-existing diseases may increase the probability of hospitalization [9]. An increased risk of acquiring severe disease was found in pregnant women with comorbidities, including those who presented with fever, cough, headache, dyspnea, and diarrhea [3]. Although a considerable fraction of pregnant women were asymptomatic, the available data were insufficient to conclude that pregnancy is not a significant risk factor for a severe clinical course.

A previous study showed that the most frequent COVID-19 neurologic symptoms among pregnant women were headache (80.8%), followed by delirium (38.4%) [11]. Neurologic complications were more frequent among critical than in non-critical COVID-19 patients [12]. Cases of Guillain–Barré syndrome [13] and cerebrovascular events were also reported among pregnant women [14]. Various mechanisms resulting in neurologic manifestations include neurologic injury from systemic dysfunction, renin-angiotensin system affectation, immune dysfunction, and direct invasion of SARS-CoV-2 of the nervous system [15,16].

There is a general paucity of data on the clinical manifestations and outcomes comparing pregnant and nonpregnant women with COVID-19 infection. The current literature is conflicting on whether pregnancy increases the risk of severe clinical outcomes in COVID-19 patients. Hence, our study aimed to determine the clinical profile of pregnant patients with COVID-19 and compare pregnant and nonpregnant women in the cohort in terms of mortality, respiratory failure, ventilator dependence, intensive care unit (ICU) admission, length of ICU stay, length of hospital stay, and neurological recovery.

## 2. Materials and Methods

### 2.1. Study Design and Participants

We performed a retrospective cohort study involving pregnant women admitted from February 2020 to December 2020 nationwide. We obtained the relevant information from the datasets of the Philippine CORONA Study in which the original study protocol and results were previously published [17,18].

### 2.2. Setting

The Philippine CORONA Study was conducted in 37 major hospitals in the Philippines [17]. The complete list of the participating hospitals is available in the published study [16].

### 2.3. Patient Selection, Sampling, and Cohort Description

All female patients with COVID-19 infection were selected from the Philippine CORONA datasets and included in the analyses. Pregnant women were grouped under the exposed cohort, while the nonpregnant women were classified under the unexposed cohort.

### 2.4. Information Sources, Data Collection, Patient Variables, and Bias

We obtained the following information: demographic/clinical profile data, comorbidities, respiratory/constitutional symptoms, neurological history, neurological symptoms/complications, and clinical outcomes, which are indicated below. With the retrospective nature of our study, recording bias is intrinsic to the data collection process.

### 2.5. Outcome Variables

The clinical outcomes considered for this study included COVID-19 severity (i.e., non-severe, severe, or critical), mortality, respiratory failure (i.e., patients requiring oxygen support with evidence of respiratory insufficiency), duration of ventilator dependence (i.e., total days the patient was hooked to assisted ventilation), ICU admission (i.e., admitted to an ICU or critical care setting), length of ICU stay (i.e., total days admitted to an ICU or critical care setting), length of hospital stay (i.e., total days admitted in the hospital), and neurological recovery (i.e., fully recovered, partially improved with residuals, or no improvement).

### 2.6. Statistical Analysis

Descriptive statistics were used, such as median and interquartile range for the age of participants in years; and time-related variables in days. Categorical variables, such as the presence of characteristics and clinical outcomes, were presented using frequency and percentage. A series of chi-square and z-tests of proportion were performed to account for differences in the distribution of characteristics and outcomes between pregnant and nonpregnant women. Wilcoxon rank-sum procedures were also conducted to compare numerical variables such as age and follow-up times across pregnancy status.

The distribution of neurological characteristics and outcomes among pregnant women was presented in tabular form using frequency and percentage. The likelihood of developing outcomes (i.e., mortality, respiratory failure, ventilator dependence, need for intensive care, ICU length of stay, and hospital length of stay) considering pregnancy status was estimated using crude and multivariable logistic regression models. The adjustment for probable confounders based on clinically important variables was also performed.

Kaplan–Meier plots were used to estimate the time-to-event outcomes considering pregnancy status. As well as further determine differences in the median survival times using a series of log-rank tests. Cox proportional hazard models were used to assess the association of pregnancy status with clinically important prognostic factors such as age, the severity of disease, new onset neurological symptoms, and the presence of conditions such as hypertension and diabetes mellitus. The adjusted and crude estimates of the hazard ratios were also computed. The significance level for all analysis sets was set at a *p*-value less than 0.05 using two-tailed comparisons. All data processing and analysis procedures were carried out using the statistical software, Stata version 13.

## 3. Results

### 3.1. Demographic Data and Clinical/ Neurological Profile of Included Patients

**Figure 1:**
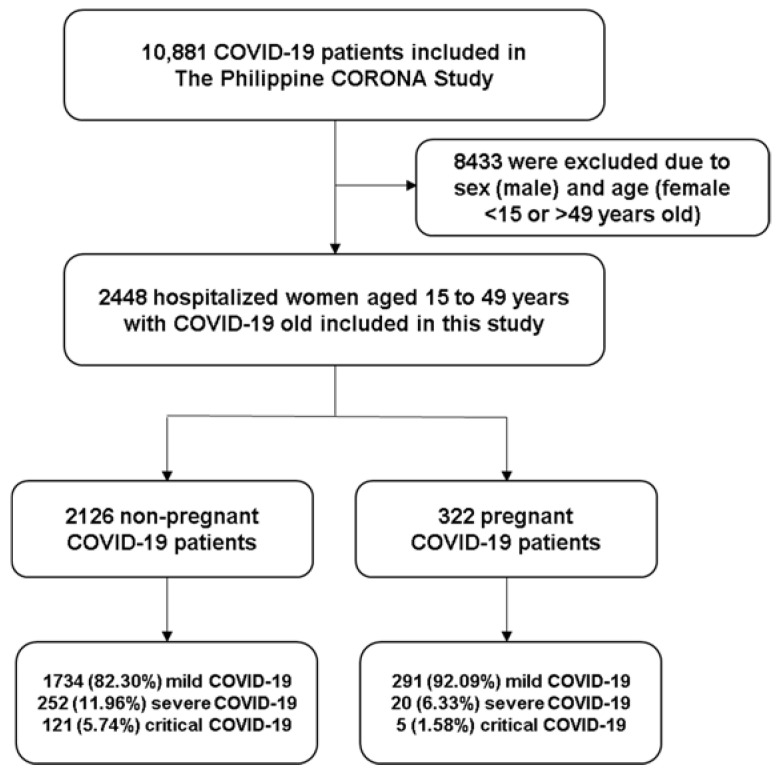
Flow of inclusion of female patient subset of the Philippine CORONA Study.

In the current study, we included 2448 women with COVID-19 from the Philippine CORONA Study database. There were 2126 (86.85%) nonpregnant and 322 (13.15%) pregnant women (Figure 1) [17]. The median age for nonpregnant and pregnant women was 33 and 30, respectively. Pregnant women in the study were significantly younger than nonpregnant participants (*p* < 0.01). The most common comorbidities were hypertension (n = 267, 10.91%), diabetes mellitus (n = 167, 6.82%), kidney disease (n = 79, 3.23%), and bronchial asthma (n = 132, 5.39%). The presence of comorbid conditions, such as hypertension (11.71% vs. 5.59%, *p* < 0.01), diabetes mellitus (7.57% vs. 1.86%, *p* < 0.01), kidney disease (3.67% vs. 0.31%, *p* < 0.01), bronchial asthma (5.93% vs. 1.86%, *p* < 0.01), was higher in nonpregnant women than pregnant women (Table 1). There were also a higher proportion of smokers, and healthcare workers among nonpregnant women, than pregnant women (*p* < 0.01). Regarding COVID-19-specific characteristics, there was a higher proportion of milder cases among pregnant women and more severe among nonpregnant study participants (*p* < 0.01).

The most common presenting symptoms in both groups were cough (n = 712, 29.1), fever (n = 619, 25.29%), and dyspnea (n = 352, 14.38%). The frequency of these symptoms was significantly higher among nonpregnant than pregnant women included in the study, as shown in (*p* < 0.05). The proportions of severe and critical cases were higher among nonpregnant (n = 373, 17.7%) than pregnant women (n = 25, 7.91%). Generally, a significantly higher percentage of nonpregnant women received COVID-19-specific interventions than pregnant women, while more than half (n = 190, 59.01%) of the pregnant women received antibiotic treatment compared with their nonpregnant counterparts (n = 641, 30.15%) (Table 1). Coincidentally, there were higher proportions of nonpregnant women who received COVID-specific interventions (i.e., systematic glucocorticoids, remdesivir, tocilizumab, lopinavir-ritonavir, and hydroxychloroquine [*p* < 0.01]). On the other hand, there was a significantly higher percentage of pregnant women who received antibiotics compared with their nonpregnant counterparts (*p* < 0.01). This can be attributed to antibiotic treatment of pregnancy-related conditions such as urinary tract infection and prophylaxis for pregnant patients with preterm prelabor rupture of membranes.

### 3.2. Comparison of Clinical/ Neurological Outcomes of Included Pregnant and Nonpregnant Women

Nonpregnant women with COVID-19 had higher mortality than pregnant women with COVID-19 (*p* < 0.01). Likewise, the development of respiratory failure (*p* < 0.01) and requirement for intensive care (*p* = 0.01) were significantly higher in nonpregnant compared with pregnant patients. There was no association between pregnancy status and reasons for intensive care, except for a higher proportion of pregnant women with cerebral edema (*p*: 0.01). The median days duration in ICU was shorter among pregnant women than nonpregnant women (*p*: 0.02). However, more than half of pregnant women remained admitted to the hospital for at least 15 days, while nonpregnant women had a shorter duration of confinement (*p* < 0.01). For the neurological outcomes, 98.15% (n = 317) of pregnant women and 93.56% (n = 1989) of nonpregnant women showed either partial improvement or complete resolution of neurological symptoms. A small proportion (n = 40, 1.88%) of nonpregnant women were clinically stable but showed no neurologic symptom improvement. In contrast, there was a higher proportion of pregnant women who were discharged, while a higher proportion of nonpregnant women died (*p* < 0.01) (Table 2).

### 3.3. Estimated Odds Ratio of Outcomes Comparing Pregnant and Nonpregnant Women (Logistic Regression Models)

Logistic regression models revealed that crude estimates for mortality (cOR: 0.26 [95% CI 0.11–0.66]; *p* < 0.01), respiratory failure (cOR: 0.37 [95% CI 0.17–0.80]; *p* = 0.01), or intensive care unit admission (cOR: 0.39 [95% CI 0.19–0.80]; *p* = 0.01) were significantly lower in pregnant patients than in nonpregnant patients with COVID-19. On the other hand, pregnant patients were more likely to stay longer in the hospital compared with nonpregnant patients (cOR: 1.73 [95% CI 1.36–2.19]; *p* < 0.01). However, statistical significance was no longer observed after adjusting for significant covariates, such as age, the severity of COVID, and the presence of new-onset neurological symptoms. Only the likelihood of hospital confinement beyond 14 days remained significant after adjusting for the covariates (aOR: 1.99 [95% CI 1.56–2.54]; *p* < 0.01) (Table 3).

### 3.4. Kaplan–Meier Plots and Estimated Hazard Ratios of Outcomes Comparing Pregnant and Nonpregnant Women (Cox Regression Models)

**Figure 2:**
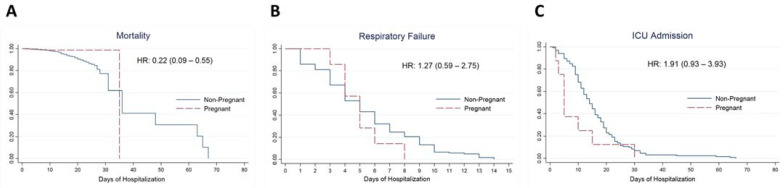
Kaplan–Meier plots comparing pregnant and nonpregnant women; (**A**), mortality; (**B**), respiratory failure; (**C**), intensive care admission.

The Kaplan–Meier plots for mortality, respiratory failure, and ICU admission are displayed in Figure 2. A series of time-to-event analyses were performed to account for the effect of pregnancy on the development of these outcomes, presented in Table 4. The unadjusted HR for mortality (HR 0.22 [95% CI 0.09, 0.55]) was significantly lower in pregnant compared with nonpregnant women. However, when adjusted for confounders, the estimate for mortality became nonsignificant. Both unadjusted and adjusted HR for respiratory failure and ICU admission were insignificant.

This lack of difference in the hazard of developing respiratory failure and ICU admission between pregnant and nonpregnant patients was further exemplified in Figure 2. No significant difference was observed in the Kaplan–Meier plots for respiratory failure and ICU admission.

## 4. Discussion

Overall, our results showed no significant difference in the disease course of COVID-19 in pregnant patients compared with nonpregnant women. Pregnant patients with COVID-19 generally were younger and had a lower prevalence of comorbidities, such as hypertension, diabetes, and kidney disease, than nonpregnant patients. A higher proportion of pregnant patients also presented with mild disease, while more severe disease was observed among nonpregnant women. Higher mortality, respiratory failure, and requirement for ICU admission were also observed in nonpregnant women compared with women with COVID-19. This finding may reflect the younger age and lower prevalence of comorbidities among pregnant women in our study. Previous reports in the Philippines also showed lower comorbidities in pregnant patients infected with COVID-19 [2,4]. Although pregnant women had a significantly more extended hospital stay, they had shorter ICU admission, shorter ventilator dependence, resolved or improved neurologic symptoms at hospital discharge, and had a lower risk of mortality. However, multivariable analyses controlling for confounders showed that pregnancy was not independently associated with most measured clinical outcomes.

Based on a local study, about 35% of COVID-19-infected pregnant women were symptomatic [2]. Similar to our result, they commonly present with fever and cough [2,3,8,19]. Compared with nonpregnant women, pregnant women more frequently present with colds and anosmia, while the former mostly present with cough, dyspnea, chills, fatigue, and headache [7]. Their risk factors include body mass index (BMI) ≥30 kg/m^2^, non-white ethnicity, living in socioeconomically deprived communities, and having comorbidities such as diabetes and hypertension [9,10,19]. Pre-existing comorbidity can increase the risk of pregnant women acquiring an infection that requires hospitalization [9]. Interestingly, a local study reported that nearly 50% of pregnant patients with COVID-19 had no comorbid condition [2]. Most of their included patients were probably under 35 years of age. There were contradicting reports on whether maternal age is a risk factor [2,5,6,9,10,19]. Theoretically, older patients are more likely to have chronic underlying diseases; thus, they are more at risk of suffering from severe to critical COVID-19.

Our results showed that the risk of severe course was significantly increased in the nonpregnant population. A higher proportion of nonpregnant women developed respiratory failure and required a longer duration of intensive care and ventilator support. A small, propensity-matched cohort study on COVID-19 revealed that nonpregnant women had a significantly higher percentage of requiring mechanical ventilation [12]. This was contrary to the findings of the meta-analysis comprising 192 studies, wherein ICU admission, invasive ventilation, and extracorporeal membrane oxygenation use were higher among pregnant individuals and recently pregnant population [19]. Their pregnant subjects had increased BMI and were more likely to have pre-existing diabetes than their nonpregnant subjects in the reproductive age group, which increased their risk of ICU admission and the need for invasive ventilation [19].

Lower thresholds for hospitalization and ICU admission were noted among pregnant women [2,9,20]. This may be attributed to the physician’s fear of developing feto-maternal morbidity and mortality in pregnant patients with COVID-19, resulting in a more aggressive treatment that led to early or unnecessary ICU admission. Healthcare providers are likely taking precautionary measures in caring for their pregnant COVID-19 patients [21]. Hence, not all pregnant patients admitted to the ICU had severe or critical COVID-19. This can potentially provide a reason for the shorter ICU stay among pregnant patients in our study. Moreover, we did not observe a significant increase in ICU admission of pregnant women than in nonpregnant women. This finding is consistent with previous studies that showed pregnant women were not at an increased risk of ICU admission compared with nonpregnant women [22,23].

Interestingly, our study showed that pregnant women were more likely to have extended hospitalization than nonpregnant women with COVID-19. Previous studies showed higher odds of hospitalization and higher mean length of stay in the hospital for pregnant women than nonpregnant women with COVID-19 [21,22]. The US Centers for Disease Control and Prevention, the American College of Obstetricians and Gynecologists (ACOG), and the Society for Maternal-Fetal Medicine (SMFM) recommended hospitalization of pregnant women diagnosed with COVID-19 in facilities with maternal and fetal monitoring capabilities when warranted and the use of multispecialty team-based approach during treatment. Furthermore, quarantine protocols and the scarcity of quarantine facilities for pregnant women are hypothesized to have influenced their extended hospitalization.

Our study showed fewer mortalities in pregnant subjects than in nonpregnant counterparts. This finding is supported by multiple studies that showed lower in-hospital death in pregnant than in nonpregnant patients [21,24,25,26]. However, factors other than pregnancy could have influenced this low mortality rate. The local medical societies in the Philippines, including the Philippine Infectious Diseases Society for Obstetrics and Gynecology (PIDSOG) and the Philippine Obstetrical and Gynecological Society (POGS), recommended routine COVID-19 screening for all near-term pregnant women, adequate prenatal care, and implementation of COVID-19 treatment guidelines. This additional attention and specialized care for pregnant patients may explain the lower morbidity and mortality rates among pregnant women in our study [27].

Our study provided extensive information on the clinical manifestation and outcomes of hospitalized pregnant women with COVID-19 compared with nonpregnant women. Compared with other published studies, the strength of our studies lies in the involvement of a relatively large number of pregnant women and detailed documentation of the clinical profile of these patients. However, this study has several limitations. Our results are based on a retrospective review of medical records known to have an inherent recording bias leading to underreporting of data. Other relevant obstetric information, such as the age of gestation and maternal and fetal outcomes, were not obtained by the initial study. Moreover, we did not obtain information on the possibility of subtle primary immunodeficiencies as a result of COVID-19 infection and treatment. Furthermore, our estimates cannot be generalized among pregnant women who were not admitted to the hospitals. Future prospective cohort studies with a rigorous methodology involving pregnant women are indispensable to verify our measured estimates. In future studies, we also recommend comparing outcomes of pregnant women based on vaccination status, during various trimesters up to the postpartum period and obtaining other relevant clinical endpoints, such as fetal and neonatal outcomes, and more detailed neurological outcomes assessments, such as using the modified Rankin scale.

## 5. Conclusions

Overall, our results suggest that pregnant women generally have better prognosis in terms of mortality, respiratory failure, ICU admission, and neurological recovery at discharge among patients admitted for COVID-19 infection. Multivariable analyses involving other confounding variables suggest that pregnancy is not an independent risk factor for these outcomes. On the contrary, pregnancy in women independently increases the risk of prolonged length of hospital stay compared with nonpregnant women in the context of the COVID-19 infection. Finally, these patients did not have prolonged ventilator dependence or ICU stay compared with nonpregnant women. Our study provides more evidence that COVID-19 infection may not result in a more severe disease progression in pregnant women than in nonpregnant women.

## Figures and Tables

**Table 1:** Clinical characteristics of the included pregnant and nonpregnant patients.

Characteristics	Overall	Nonpregnant	Pregnant	*p* Value
Frequency	2448	2126 (86.85%)	322 (13.15%)	-
Age in years	33 (13)	33 (14)	30 (9)	
15–20 years	64 (2.61%)	40 (1.88%)	24 (7.45%)	<0.01 *
21–30 years	957 (39.09%)	803 (37.77%)	154 (47.83%)	
31–40 years	827 (33.78%)	697 (32.78%)	130 (40.37%)	
41–49 years	600 (24.51%)	586 (27.56%)	14 (4.35%)	
Presence of comorbid conditions				
Hypertension	267 (10.91%)	249 (11.71%)	18 (5.59%)	<0.01 *
Diabetes mellitus	167 (6.82%)	161 (7.57%)	6 (1.86%)	<0.01 *
Kidney disease	79 (3.23%)	78 (3.67%)	1 (0.31%)	<0.01 *
Bronchial asthma	132 (5.39%)	126 (5.93%)	6 (1.86%)	<0.01 *
Coronary artery disease	19 (0.78%)	19 (0.89%)	-	0.16
Malignancy	41 (1.67%)	41 (1.93%)	-	0.01 *
COPD	1 (0.04%)	-	1 (0.31%)	0.13
Heart failure	6 (0.25%)	5 (0.24%)	1 (0.31%)	0.80
Liver disease	6 (0.25%)	5 (0.24%)	1 (0.31%)	0.80
Immunocompromised conditions	3 (0.12%)	3 (0.14%)	-	0.50
Obesity	22 (0.90%)	21 (0.99%)	1 (0.31%)	0.23
Other conditions	309 (12.62%)	289 (13.59%)	20 (6.21%)	<0.01 *
Relevant history				
Smoker	55 (2.25%)	55 (2.59%)	-	<0.01 *
Healthcare worker	393 (16.05%)	387 (18.20%)	6 (1.86%)	<0.01 *
COVID-19 disease severity				
Mild	2025 (83.57%)	1734 (82.30%)	291 (92.09%)	<0.01 *
Severe	272 (11.23%)	252 (11.96%)	20 (6.33%)	
Critical	126 (5.20%)	121 (5.74%)	5 (1.58%)	
Respiratory and constitutional symptoms				
Cough	712 (29.08%)	669 (31.47%)	43 (13.35%)	<0.01 *
Fever	619 (25.29%)	598 (28.13%)	21 (6.52%)	<0.01 *
Dyspnea	352 (14.38%)	337 (15.85%)	15 (4.66%)	<0.01 *
Sore throat	198 (8.09%)	191 (8.98%)	7 (2.17%)	<0.01 *
Fatigue	112 (4.58%)	108 (5.08%)	4 (1.24%)	<0.01 *
Sputum production	94 (3.84%)	88 (4.14%)	6 (1.86%)	0.05 *
Rhinorrhea	182 (7.43%)	169 (7.95%)	13 (4.04%)	0.01 *
Diarrhea	136 (5.56%)	132 (6.21%)	4 (1.24%)	<0.01 *
Arthralgia	34 (1.39%)	32 (1.51%)	2 (0.62%)	0.21
Hemoptysis	1 (0.04%)	1 (0.05%)	-	0.70
Treatment Received				
Systematic glucocorticoids	314 (12.83%)	300 (14.11%)	14 (4.35%)	<0.01 *
Remdesivir	132 (5.39%)	129 (6.07%)	3 (0.93%)	<0.01 *
Tocilizumab	65 (2.66%)	63 (2.96%)	2 (0.62%)	<0.01 *
Lopinavir-Ritonavir	64 (2.61%)	64 (3.01%)	-	<0.01 *
Hydroxychloroquine	112 (4.58%)	110 (5.17%)	2 (0.62%)	<0.01 *
Chloroquine	38 (1.55%)	37 (1.74%)	1 (0.31%)	0.06
Convalescent plasma	21 (0.86%)	21 (0.99%)	-	0.10
Antibiotic therapy	831 (33.95%)	641 (30.15%)	190 (59.01%)	<0.01 *
Other regimens	600 (24.51%)	527 (24.79%)	73 (22.67%)	0.41

Abbreviations: COPD: chronic obstructive pulmonary disease; COVID-19: coronavirus disease. * Statistically significant (i.e., *p*-value less than 0.05).

**Table 2:** Distribution of clinical outcomes among women in the study population.

Outcomes	Overall	Nonpregnant	Pregnant	*p*-Value
Frequency	2448	2126 (86.85%)	322 (13.15%)	-
Mortality and associated causes				
Mortality	123 (5.02%)	118 (5.55%)	5 (1.55%)	<0.01 *
Acute respiratory distress syndrome	55 (44.72%)	52 (44.07%)	3 (60%)	0.48
Septic shock	35 (28.46%)	32 (27.12%)	3 (60%)	0.11
Multi-organ dysfunction syndrome	14 (11.38%)	14 (11.86%)	-	0.41
Acute coronary syndrome	7 (5.69%)	7 (5.93%)	-	0.58
Cardiac arrhythmia	5 (4.07%)	5 (4.24%)	-	0.64
Brain herniation	7 (5.69%)	7 (5.93%)	-	0.58
Decompensated heart failure	1 (0.81%)	1 (0.85%)	-	0.84
Other causes	31 (35.20%)	29 (24.58%)	2 (40%)	0.44
Respiratory failure and causes				
Respiratory failure	128 (5.23%)	121 (5.69%)	7 (2.17%)	0.01 *
Pneumonia	71 (55.47%)	67 (55.37%)	4 (57.14%)	0.93
Acute respiratory distress syndrome	58 (45.31%)	56 (46.28%)	2 (28.57%)	0.36
Shock	9 (7.03%)	8 (6.61%)	1 (14.29%)	0.44
Central neurological cause	4 (3.13%)	4 (3.31%)	-	0.63
Pulmonary edema	2 (1.56%)	2 (1.65%)	-	0.73
Pulmonary embolism	5 (3.91%)	5 (4.13%)	-	0.58
Duration of ventilator dependence	11 (10)	12 (9)	5 (11)	0.20
1–5 days	75 (58.89%)	70 (57.85%)	5 (71.43%)	0.48
6 or more days	53 (41.41%)	51 (42.15%)	2 (28.57%)	
ICU Admission and reasons				
Needed intensive care	139 (5.68%)	131 (6.16%)	8 (2.48%)	0.01 *
Respiratory failure	84 (60.43%)	78 (59.54%)	6 (75%)	0.39
Acute respiratory distress syndrome	63 (45.32%)	61 (46.56%)	2 (25%)	0.23
Shock	13 (9.35%)	11 (8.40%)	2 (25%)	0.12
Impaired level of consciousness	12 (8.63%)	10 (7.63%)	2 (25%)	0.09
Acute myocardial infarction	4 (2.88%)	4 (3.05%)	-	0.62
Acute kidney injury	10 (7.19%)	9 (6.87%)	1 (12.50%)	0.55
Treatment-related indication	13 (9.35%)	11 (8.40%)	2 (25%)	0.12
Acute stroke	5 (3.60%)	4 (3.05%)	1 (12.50%)	0.16
Cardiac arrhythmia	2 (1.44%)	2 (1.53%)	-	0.73
Post-cardiac arrest	2 (1.44%)	2 (1.53%)	-	0.73
Cerebral edema	2 (1.44%)	1 (0.76%)	1 (12.50%)	0.01 *
Venous thromboembolism	1 (0.72%)	1 (0.76%)	-	0.80
Time to ICU Admission	5 (4)	5 (4)	5 (4)	0.65
Duration of ICU Stay	14 (11)	14 (11)	5 (8)	0.02 *
1–7 days	80 (57.55%)	73 (55.73%)	7 (87.50%)	0.08
8 or more days	59 (42.45%)	58 (44.27%)	1 (12.50%)	
Duration of Hospital Stay	13 (8)	13 (7)	14 (10)	<0.01 *
1–14 days	1353 (55.27%)	1213 (57.06%)	140 (43.48%)	<0.01 *
15 or more days	1095 (44.73%)	913 (42.94%)	182 (56.52%)	
Neurologic Outcomes				
Full recovery	1973 (80.60%)	1762 (82.88%)	211 (65.53%)	<0.01 *
Stable with improvement	333 (13.60%)	227 (10.68%)	106 (32.92%)	
Stable without improvement	40 (1.63%)	40 (1.88%)	-	
Not applicable	102 (4.17%)	97 (4.56%)	5 (1.55%)	
Final Disposition				
Discharged	2308 (94.28%)	1991 (93.65%)	317 (98.45%)	<0.01 *
HAMA	17 (0.69%)	17 (0.80%)	-	
Died	123 (5.02%)	118 (5.55%)	5 (1.55%)	

Abbreviations: HAMA: home against medical advice; ICU: intensive care unit. * Statistically significant (i.e., *p*-value less than 0.05).

**Table 3:** Association of pregnancy and select clinical outcomes.

Outcomes	OR (95% CI)	*p*-Value
Mortality		
Crude estimates for pregnancy	0.26 (0.11–0.66)	<0.01 *
Adjusted for age, disease severity, and NNS	0.51 (0.16–1.68)	0.27
Respiratory Failure		
Crude estimates for pregnancy	0.37 (0.17–0.80)	0.01 *
Adjusted for age, disease severity, and NNS	0.89 (0.32–2.49)	0.82
Ventilator dependence (>5 days)		
Crude estimates for pregnancy	0.55 (0.10–2.94)	0.48
Adjusted for age, disease severity, and NNS	0.88 (0.14–5.56)	0.90
Need for Intensive Care		
Crude estimates for pregnancy	0.39 (0.19–0.80)	0.01 *
Adjusted for age, disease severity, and NNS	0.90 (0.39–2.11)	0.81
ICU Length of Stay (>7 days)		
Crude estimates for pregnancy	0.18 (0.02–1.50)	0.11
Adjusted for age, disease severity, and NNS	0.15 (0.02–1.39)	0.09
Hospital Length of Stay (>14 days)		
Crude estimates for pregnancy	1.73 (1.36–2.19)	<0.01 *
Adjusted for age, disease severity, and NNS	1.99 (1.56–2.54)	<0.01 *

* Adjusted for age, disease severity, and new-onset neurological symptoms. Abbreviations: NNS: new-onset neurological symptoms; ICU: intensive care unit.

**Table 4:** Time to event analysis for select outcomes and pregnancy.

Outcomes	HR (95% CI)	*p*-Value
Time to Mortality		
Crude estimates for pregnancy	0.22 (0.09–0.55)	<0.01 *
Adjusted for age, disease severity, NNS, hypertension, diabetes	0.65 (0.26–1.64)	0.35
Time to Respiratory Failure		
Crude estimates for pregnancy	1.27 (0.59–2.75)	0.54
Adjusted for age, disease severity, NNS, hypertension, diabetes	1.54 (0.68–3.49)	0.30
Time to ICU Admission		
Crude estimates for pregnancy	1.91 (0.93–3.93)	0.08
Adjusted for age, disease severity, NNS, hypertension, diabetes	2.01 (0.94–4.29)	0.07

* Adjusted for age, disease severity, new-onset neurological symptoms, hypertension, and diabetes mellitus. Abbreviations: NNS: new-onset neurological symptoms.

## Data Availability

Anonymized data not published within this article will be made available by request from any qualified investigator.
